# Current evidence and outcomes for retinopathy of prematurity prevention: insight into novel maternal and placental contributions

**DOI:** 10.37349/emed.2020.00002

**Published:** 2020-02-29

**Authors:** Lara Carroll, Leah A. Owen

**Affiliations:** Department of Ophthalmology and Visual Sciences, University of Utah, Salt Lake City, UT 4132, USA

**Keywords:** Retinopathy of prematurity, prevention, therapeutics, placenta

## Abstract

Retinopathy of prematurity (ROP) is a blinding morbidity of preterm infants, which represents a significant clinical problem, accounting for up to 40% of all childhood blindness. ROP displays a range of severity, though even mild disease may result in life-long visual impairment. This is complicated by the fact that our current treatments have significant ocular and potentially systemic effects. Therefore, disease prevention is desperately needed to mitigate the life-long deleterious effects of ROP for preterm infants. Although ROP demonstrates a delayed onset of retinal disease following preterm birth, representing a potential window for prevention, we have been unable to sufficiently alter the natural disease course and meaningfully prevent ROP. Prevention therapeutics requires knowledge of early ROP molecular changes and risk, occurring prior to clinical retinal disease. While we still have an incomplete understanding of these disease mechanisms, emerging data integrating contributions of maternal/placental pathobiology with ROP are poised to inform novel approaches to prevention. Herein, we review the molecular basis for current prevention strategies and the clinical outcomes of these interventions. We also discuss how insights into early ROP pathophysiology may be gained by a better understanding of maternal and placental factors playing a role in preterm birth.

## Introduction

Retinopathy of prematurity (ROP) represents a significant clinical problem accounting for up to 40% of childhood blindness worldwide with an estimated incidence of 68% in infants born less than 1,250 g [1–3]. With increasing viability of earlier gestational age (GA) infants worldwide, the incidence of ROP is increasing prompting concern for a “third epidemic”, particularly in middle or low-income countries [4]. ROP ranges in severity, though even mild disease that clinically regresses is associated with long-term visual impairments [5–8]. Thus, the scope of the problem is increasing, making the life-long burden of blindness in this population an even greater clinical concern.

ROP is a disorder of retinal vascular maturation with unclear molecular etiology; early birth, low birth weight (BW), and post-natal oxygen exposure are the only universally accepted independent disease predictors [9–12]. In humans, full retinal vascularization does not developmentally occur until approximately 36–40 weeks corrected GA. Therefore, when born preterm, retinal vascularization is incomplete and must occur in an environment dissimilar to the in-utero environment. ROP represents aberrancy in this process and occurs in two phases. The initial phase occurs prior to 34 weeks GA and is defined by relative hyperoxia and arrest of retinal vascularization. The second phase occurs after 34 weeks and is defined by retinal hypoxia and the manifestation of clinical retinal disease, chiefly pre-retinal neovascularization at the junction of vascularized and a vascular retina [9, 10, 13]. Thus, only the second phase of ROP demonstrates retinal neovascular disease and potentially permanent changes in retinal architecture.

On the basis of this understanding, intervention in the “pre-clinical” phase 1 disease would allow for facilitation of normal retinal vascularization and prevent progression to the second phase of ROP. This necessitates an understanding of early ROP risk and disease mechanisms, occurring prior to and within the first phase of ROP. While overall these are incompletely understood, there has been a significant effort to better delineate early ROP pathological mechanisms [10, 11, 14–16]. Work from our group and others substantiates that only low BW, prematurity and post-natal oxygen exposure confer independent ROP risk [9–11, 15, 17–19]. Our current screening metrics, based on this understanding of risk, include GA at birth and BW; however, the specificity of these metrics is only approximately 50% [9, 10]. Further, while post-natal oxygen is the most modifiable risk, limiting oxygen increases infant mortality [9, 20, 21]. As a result, our screening is imprecise and current interventions are unable to modify risk and instead, target ROP once retinal disease is present and we are unable to restore normal retinal architecture and visual function [22, 23]. Further, treatment is indicated only in advanced disease with a high probability of retinal detachment and therefore the goal of treatment is largely to prevent this outcome. While our interventions do often achieve this goal, current gold standard treatments are associated with significant ocular and visual morbidity, including retinal scarring, loss of peripheral vision, degenerative myopia and cataract. Therefore, there is significant clinical need for novel understanding of early ROP risk and disease mechanisms to increase screening specificity and prevent retinal disease, facilitating normal retinal vascularization.

Herein, we review key early molecular disease pathogenesis that has informed treatment and prevention therapies, clinical findings based on these interventions, novel insights from maternal, and placental pathobiology to identify ROP disease prevention strategies.

## Established ROP patho-mechanisms

While ROP early risk and mechanisms are incompletely understood, we have greater understanding of the “final common pathway” mediating neovascular retinal disease.

Studies have shown that birth and the consequent switch to lung ventilation produces a change in arterial blood oxygen tension significantly above that of the intrauterine fetus [24]. In the incompletely vascularized preterm infant retina, this “supra-physiologic” tissue oxygenation is felt to initiate the first phase of ROP. During this hyperoxic phase, angiogenic factors such as vascular endothelial growth factor (VEGF) and growth factors such as insulin growth factor 1 (IGF-1) are down-regulated. These molecular changes correlate with arrest of retinal vascularization and pruning of newly formed vasculature leading to retinal avascularity. This process is potentiated by the necessary use of supplemental oxygen, which may also contribute to oxygen toxicity when the production of reactive oxygen species exceeds the capacity of the neonate’s antioxidant defense mechanisms, causing apoptosis and further regression of newly formed vessels [25–27]. When the pre-term infant reaches approximately 32–34 weeks, the growing retina experiences an upward shift in its metabolic demands, prompting the second phase of ROP defined by a period of relative hypoxia [13, 15]. In this context, retinal astrocytes and Muller glia have been shown to up-regulate production of VEGF, IGF-1 and other growth factors [28], resulting in the appearance of proliferative neovascularization characterized by growth of disorganized leaky capillaries into the vitreous.

**Figure 1.**
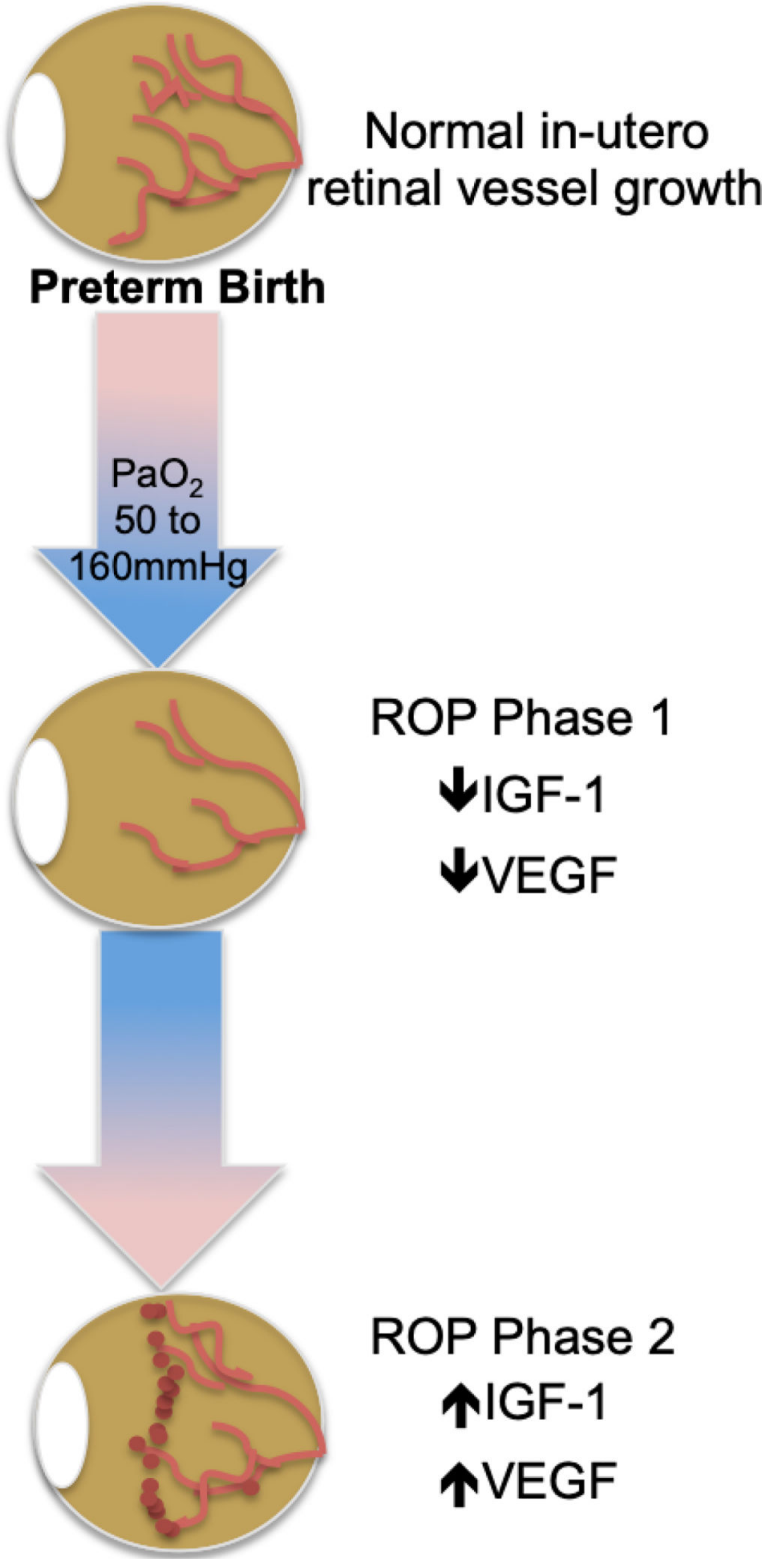
ROP molecular pathogenesis. ROP occurs in two phases following preterm birth and is mediated by a) oxygen; b) VEGF; and c) IGF-1

This neovascular event has the potential for greatest visual harm as the vitreally-directed vessel growth can result in tractional retinal detachment and irreversible blindness. The central events and molecular changes that form the framework of ROP pathology, as summarized in Figure 1, are the basis for current treatment modalities and for some emerging prevention therapies.

### ROP molecular pathogenesis informs current ROP interventions

Fluctuations in angiogenic growth factors occurring in the preterm retina and mediating ROP disease are thought to primarily arise from avascular retina [13]. This understanding forms the basis for our current therapeutic interventions, which are aimed at amelioration of neovascularization in the second phase of ROP. These include ablation of avascular retinal tissues, now done with laser, or reduction in expression of the proangiogenic growth factor VEGF and can be administered as a monotherapy or as sequential therapy [29–31].

#### Anti-VEGF treatment for ROP

Early studies in animal models of oxygen induced retinopathy (OIR) showed that supplemental VEGF at the onset of hyperoxia significantly decreased both initial vaso-obliteration and the subsequent neovascularization characteristic of ROP, and that inhibition of VEGF was a viable treatment for neovascular disease once established [32–34]. These animal findings were substantiated in humans when use of Avastin, a humanized anti-VEGF monoclonal antibody that had proven effective for pathological retinal neovascularization in adults, was first shown to be efficacious in treatment of ROP [35–37]. However, there is concern for potential adverse local and systemic effects of intraocular anti-VEGF treatments [38–43]. VEGF is now recognized as an important trophic factor for a variety of retinal cell types including retinal neurons, RPE, and glia [44, 45].The potential for alteration in the development of these retinal cells by broad-based VEGF blockage requires further study in the developing eye. Moreover, the potential leakage of anti-VEGF into systemic circulation warrants caution regarding the potentially adverse effects of suppressing VEGF required for extra-ocular physiologic processes [46–49]. Despite the growing use of anti-VEGF therapy in ROP and its support from the American Academy of Ophthalmology and the American Academy of Pediatrics [50], there remains a paucity of information regarding systemic and local safety and efficacy of anti-VEGF therapeutics in ROP disease. Additional controlled clinical studies are needed to provide necessary guidance for optimizing dosage and timing of anti-VEGF injections in preterm newborns for improved standard of care [51].

#### Ablative therapy for ROP

Cryotherapy and laser photocoagulation are considered the gold standard ROP treatment [22, 23, 52, 53]. Both methods achieve regression of neovascular retinal disease through ablation of the peripheral avascular retina, eliminating cellular oxygen demand and thereby reducing production of VEGF and other ischemic tissue factors. These treatments greatly reduce neovascularization; nevertheless, ablative treatments can lead to significant peripheral and central vision loss and are further associated with increased risk of cataract and degenerative myopia. Therefore, while these treatments do prevent catastrophic loss of retinal architecture, function, and thus vision, they are associated with significant visual morbidity.

Taken together, our current therapeutic interventions have significant limitations, side-effects, and systemic concerns. Furthermore, they largely lack the ability to restore normal retinal architecture and function through preservation of physiologic retinal vascularization. To this end, work has been done to elucidate mechanisms informing early ROP pathogenesis, within the first phase of ROP, which would allow for intervention prior to development of neovascular disease. These interventions, outcomes and potential for further advancement are reviewed below.

### Preventive interventions informed by ROP molecular pathogenesis

#### Oxygen modulation

Gestational age, BW, and post-natal oxygen supplementation are well-established to be the three greatest risk factors independently informing ROP risk [10, 17]. Of these risk variables, oxygen exposure is the most modifiable, however modulation of post-natal oxygen has been associated with increased mortality [20, 21, 54] (reviewed by Owen and Hartnett, 2014). Therefore, while increased post-natal oxygen is important for preterm infant neurovascular and lung development, it is deleterious for retinal vascular development. This inverse relationship has overall been prohibitive for ROP prevention strategies based on absolute reductions in post-natal oxygen administration. Despite this, clinical studies continue to examine oxygen variables, such as fluctuation in oxygen saturation, timing, duration, and percent oxygen delivery to establish optimal treatments that can be accommodated to individual infant needs. Although such efforts are gradually delineating the best practices for maximizing preterm infant visual outcomes, there is yet no clinical consensus regarding protocols and target ranges for preterm oxygen therapy, which is particularly true in developing countries with the greatest disease burden [4, 54–56]. Various co-morbidities that impact systemic oxygenation such as bronchopulmonary dysplasia [57, 58], thrombocytopenia [59], and anemia [60, 61] are also associated with ROP development and/or severity. Interventions targeting these co-morbid conditions, such as transfusion, often show association with more severe ROP disease, though this is likely a surrogate indicator of hypoxia as the etiologic factor [62]. Taken together, data suggest that regulating oxygen alone for co-morbid conditions that contribute to hypoxia does not represent a comprehensive solution for ROP prevention.

#### Supplemental IGF-1

IGF-1 is a growth factor critical for normal vascular development, provided in part by the placenta and amniotic fluid during gestation. After term birth, neonate serum levels of IGF-1 decrease; this change is exacerbated in the setting of preterm birth [63]. Further, premature infants who develop ROP have lower serum levels of IGF-1 at birth compared to age-matched infants without disease, suggesting that low IGF-1 is an independent risk factor for subsequent ROP disease [64–67]. However, post-natal growth rate proved to be a suitable proxy for IGF-1 levels, and has largely replaced IGF-1 for modeling ROP risk. On a molecular level, decreased IGF-1 has been shown to result in impaired VEGF-mediated endothelial survival and therefore, links IGF-1 function to fundamental ROP pathophysiology [68]. This is supported by rodent studies demonstrating that pharmacological inhibition of IGF-1 prevents VEGF pathway activation, oxygen-mediated vessel loss, and subsequent neovascularization in the OIR model of ROP, suggesting a permissive role of IGF-1 in VEGF-mediated retinal neovascularization [69, 70]. These data have informed current clinical studies to determine if replenishing IGF-1 in preterm infants is preventive for subsequent neovascular proliferation [71]. In a small pilot trial, preterm infants were treated with a continuous infusion of recombinant human IGF-1 (rhIGF-1) and its receptor IGF binding protein-3 (rhIGFBP-3) which demonstrated increased IGF-1 and IGFBP-3 serum concentrations without safety concerns [72]. However, recently published Phase II clinical trials reported no reduction in ROP occurrence or severity after supplemental human rhIFG-I/rhIGFBP-3 administration [73]. As noted by the authors, the IGF-1 treatment effect may be unavoidably masked by high oxygen supplementation; however, as supplemental oxygen continues to be an important part of clinical care, IGF-1 supplementation does not appear to provide a viable monotherapy for ROP prevention. Interestingly, severe bronchopulmonary dysplasia was significantly reduced, and intraventricular hemorrhage showed a downward trend, suggesting that there may be utility to further study of IGF-1 supplementation in preterm neonates. This is substantiated by literature demonstrating a potential role for IGF-1 therapy in treatment of neurodegenerative disease. Specifically, a recent meta-analysis and systematic review demonstrates positive effects of IGF-1 administration on neuroregeneration in animal models, though clinical study data for human conclusions is limited [74]. Therefore, IGF-1 may play a greater role in future efforts to normalize retinal development.

#### HIF-1 stabilization

Hypoxia-inducible factor 1 (HIF-1) is a transcription factor recognized as a master regulator of oxygen response under developmental, physiologic, and pathologic conditions [75, 76]. Under low systemic oxygen, the two HIF-1 subunits, HIF-1α, and HIF-1β, are up-regulated and initiate transcription of hypoxia-responsive genes, inducing VEGF and other angiogenic factors, prompting vasculogenesis, angiogenesis, and red blood cell production to facilitate increased oxygen delivery to ischemic tissue. On the basis of this molecular function, stabilization of HIF levels during the hyperoxic phase of ROP may prevent initial vascular attenuation and the consequent tissue hypoxia responsible for subsequent neovascular disease. This strategy has been tested in rodents using the OIR model of pre-retinal neovascularization and ROP, with systemic pharmacologic stabilization of Hif-1α with dimethyloxalylglycine or Roxadustat. Collectively, these studies demonstrated a significant reduction in both avascular retinal area and intravitreal neovascularization relative to control, with no reported adverse effects [77–79]. Based on these data, timely systemic stabilization of HIF-1 may be an effective strategy to promote peripheral vascular development and prevent ROP. However, this approach has limitations when applied to preterm infants; namely, precise therapeutic timing at the onset of hyperoxia is critical as HIF-1 stabilization in the presence of retinal hypoxia may have the undesired effect of exacerbating VEGF-mediated proliferative vascularization [80]. Thus, the opposite therapeutic approach, HIF-1 inhibition (rather than stabilization), is being interrogated as a means to limit VEGF-induced vascularization during the second phase of ROP. In a recent report, topotecan, and doxorubicin, two drugs shown to mediate HIF-1 inhibition through alternate mechanisms, were both found to reduce neovascular tuft formation in mice after systemic administration during the second, hypoxic, phase of the OIR model [81, 82]. These interventions are promising, though prevention approaches are currently limited by incomplete understanding of optimal timing of HIF-1 stabilization and lack of translational human studies.

#### Caffeine

Caffeine was first observed to reduce severe ROP outcomes in a randomized multicenter clinical trial examining neurodevelopment and growth in preterm infants treated for apnea of prematurity. At 18–21 months, the surviving caffeine treated infants had significantly less severe ROP than placebo infants, with no difference in mortality [83]. Two recent meta-analyses similarly reported a reduction of ROP risk after caffeine treatment in preterm neonates [84, 85]. This effect has been explored in mouse and rat models of OIR [86], which have demonstrated a caffeine-associated decrease in both avascular area and neovascularization of mouse retinas undergoing hyperoxia, supporting a preventive paradigm for caffeine. Similarly, in a rat OIR model, caffeine treatment reduced retinal neovascularization, while co-treatment with topical eye drops containing the non-steroidal anti-inflammatory drug (NSAID) ketorolac, further decreased severe angiogenesis [87]. The molecular basis that informs this therapeutic effect is not completely clear and may in part be related to therapeutic NSAID effects [88]. However, caffeine appears to manipulate key ROP molecular pathogenesis. For example caffeine has been shown to regulate endothelial apoptosis and angiogenesis by regulating levels of HIF1α, VEGF, and IGF-1, and by decreasing inflammatory pathways through reduction of NF-kB cyclooxygenase (Cox-2) activity [86, 87, 89]. Early caffeine administration, within the first 2 post-natal days, was recently found to be associated with improved neurodevelopmental outcomes, though studies designed to understand independent prevention or treatment significance fail to demonstrate a role for caffeine administration in ROP management [10, 17, 90]. Therefore, caffeine alone does not appear to offer sufficient ROP prevention.

#### Beta blockers

Propranolol, a nonselective beta-adrenoreceptor blocker (B-AR), was initially proposed as a treatment for neovascular ROP disease after it was found effective in reducing the growth of infantile capillary hemangiomas, which demonstrate VEGF-mediated pathogenesis similar to ROP [91]. Propranolol is thought to modulate VEGF function through activation of the B2 and B3 adrenoreceptors, which results in attenuation of vascularization through inhibition of norepinephrine-mediated elevations in HIF-1, IGF-1, and VEGF [92–96]. The effectiveness of propranolol for both prevention [95] and treatment [96–99] of pre-retinal neovascularization in the murine OIR model demonstrates mixed results, though the preponderance of evidence suggests efficacy. However, studies in preterm infants have raised concern for both efficacy and safety. Although oral propranolol showed promise in halting ROP progression, there was an increase in adverse life-threatening side-effects in pilot clinical trials [100], and a recent multi-center phase IIB study using 0.2% propranolol in newborns with stage 1 ROP reported that despite overall favorable treatment results, early treatment resulted in worse ROP disease, suggesting that a β-adrenoreceptor blockade is only useful during the proliferative phase [101]. These findings may harmonize the disparate data with regard to efficacy of propranolol for ROP treatment, but nevertheless raise concern for preventive β-adrenoreceptor inhibition in ROP. Further, recent studies demonstrate a possible deleterious refractive outcome of topical propranolol therapy, highlighting the importance of continued study [102]. Conversely, a recent meta-analysis including clinical trial and study data for 461 infants, demonstrated efficacy of oral propranolol for ROP prevention in preterm infants demonstrating a decreased relative risk of severe ROP disease (not complete ROP prevention), supporting prior PreROP trial data [103, 104]. However the authors do note that additional well-powered, multinational, randomized control trials reporting on long-term outcomes are needed.

#### Human breast milk

Perinatal inflammation [105–107] and nutritional deficiencies [108–110] are postulated to render the developing retina more susceptible to ROP pathogenesis. Nutritional deficiencies in polyunsaturated fatty acids (PUFAs), inositol, vitamins A, E, and D supplied in human breast milk (BM) are known to increase inflammation and oxidative stress and therefore these processes are likely inter-related. Increased levels of inflammatory markers have been identified in placentas from preterm births associated with increased ROP risk, as well as in newborn and maternal serum from such pregnancies [105]. Further, the contribution of global nutritive status, as measured by post-natal weight gain, has an increasingly recognized contribution to early ROP risk; models incorporating these factors into risk stratification can improve prediction accuracy for ROP, ultimately reducing the number of preterm neonates subjected to eye exams [19, 111–114]. Human BM contains immunoglobulins, digestive enzymes, important nutrients such as docosahexaenoic acid (DHA) and inositol, as well as maternally transferred vitamins such as vitamins A, C, D, E, and K, riboflavin and niacin, and most studies show a decreased association of ROP in preterm infants fed mothers milk [115–118]. However there may be nutritional differences in preterm and term BM that can affect clinical outcomes [108]. We are beginning to quantify these differences and optimize our abilities to provide preterm infants with key nutritional components in BM [119, 120]. With increased understanding of nutritional and inflammatory differences in the preterm neonate, as well as the interplay between these factors, we will be better able to understand the preventive role for BM supplementation in ROP pathogenesis. Based on current evidence, this is not a viable prevention strategy at present.

#### Vitamin E supplementation

The first nutritional and antioxidant supplement to be tested in preterm neonates for improving ROP outcome was vitamin E [121]. However, in the 70 years since its off-label use, a multitude of observational and controlled clinical studies have not clearly delineated benefits of vitamin E supplementation for treating ROP. Although an active clinical trial is currently interrogating the efficacy of a topical formulation of vitamin E plus other antioxidants [122], vitamin E supplementation is no longer considered a viable strategy for ROP prophylaxis, particularly amidst reports of increased morbidity.

#### Inositol supplementation

Work has also been done to investigate the role for non-maternal milk sources of dietary supplements to improve preterm neonate health and prevent development of ROP, though there are mixed results. For instance, while two early clinical studies testing inositol supplementation reported an associated decrease in ROP incidence [123, 124], a recent randomized clinical trial was terminated early after a trend of increased mortality with treatment, and recent systematic reviews and meta-analyses were unable to detect a significant effect of inositol on preventing ROP [125–127].

#### Vitamin A supplementation

Vitamin A supplementation demonstrates greater promise for ROP prophylaxis. Systemic concentrations of the vitamin A metabolite retinol are low in preterm infants [128, 129], and observational studies have demonstrated a reduced risk of ROP with aggressive supplementation of vitamin A [130]. The mechanistic role of vitamin A relative to fundamental ROP pathogenesis is not clear, though there is precedent for the importance of vitamin A metabolism for photoreceptor function, retinal health and vision. Specifically, preterm neonates showed improved dark-adapted rod sensitivity when supplemented with high-dose Vitamin A [131]. Moreover, results from the first randomized control trial testing vitamin A supplementation for ROP prevention demonstrated that supplementation with 1,500 IU/day vitamin A was associated with significantly lower risk of Type 1 ROP compared to the control group, with no reported signs of vitamin A toxicity [132]. While certainly promising, vitamin A supplementation did not reduce overall ROP development and therefore, likely does not represent a singular preventive approach.

#### Polyunsaturated fatty acid supplementation

Some of the most promising and least contentious supplements currently in clinical trials are the ω−3 long chain PUFAs DHA and eicosapentaenoic acid (EPA). Both have well established benefits in protecting against inflammation and oxidative stress contributing to neurodegeneration [133]. Moreover, these fatty acids play a role in modulating IGF-1 activation [134] and VEGF-mediated endothelial signaling [135,136], and may therefore contribute directly to ROP molecular pathogenesis. Animal studies overall support a role for fatty acid supplementation as treatment and possibly prevention for ROP. Supplementation with DHA and EPA were found to reduce retinal avascular area in mice subjected to hyperoxia, partly by suppression of proinflammatory tumor necrosis factor-α which itself promotes angiogenesis via cooperation with VEGF [137]. Furthermore, dietary omega-3 fatty acid supplementation during the proliferative stage of OIR also improved retinal outcome, greatly reducing neovascularization [138]. However, Beharry et al. [139], found a higher incidence of peripheral hemorrhaging in omega-3 treated pups, possibly due to the anti-thrombotic effects of PUFAs [140]. ROP outcomes after long-chain polyunsaturated fatty acid supplementation have been tested in preterm infants with largely favorable results. In three randomized control trials and a small observational study of very low BW infants, fish oil supplementation (which contains both DHA and EPA) was found to significantly decrease ROP risk or severity [141–144]. A subsequent systematic review and meta-analysis comparing fish-oil lipid emulsions with soybean-based lipid emulsions, reported a significantly reduced risk of severe ROP or ROP requiring laser therapy after the fish-oil supplement, suggesting that omega-3 PUFAs may help prevent development of aggressive ROP [145]. However, no ROP effect was measured in two recent randomized clinical trials testing supplementary fish oil or a mixed lipid emulsion containing soybean oil, medium chain triglycerides, olive oil and fish oil compared to control treatment [142, 146]. In one of two recent randomized clinical trials testing DHA supplementation alone, a decrease in stage 3 (severe) ROP was reported, though neither study found a reduction in overall ROP incidence [147, 148]. These conflicting results may be explained by, a recent study which found that a high ratio of preterm infant erythrocyte omega-6/omega-3 PUFA (ratio of arachidonic acid to DHA) at birth, positively correlated with later development of ROP [149], suggesting that the ratio of omega-6/omega-3 PUFAs rather than absolute levels of omega-3s in blood may be a better predictor of ROP risk. Additional studies are needed to examine the implications of PUFA ratios in a clinical setting, though greater understanding of effective dosing, timing and fatty acid composition may lead to preventive supplementation in the future. A randomized, controlled clinical trial due to be completed in December 2019 testing intravenous omega-3 fatty acid supplementation for ROP prevention will additionally report infant peripheral circulation lipidomic and transcriptomic profile outcomes and thus, may contribute to these knowledge gaps (NCT02486042).

**Figure 2.**
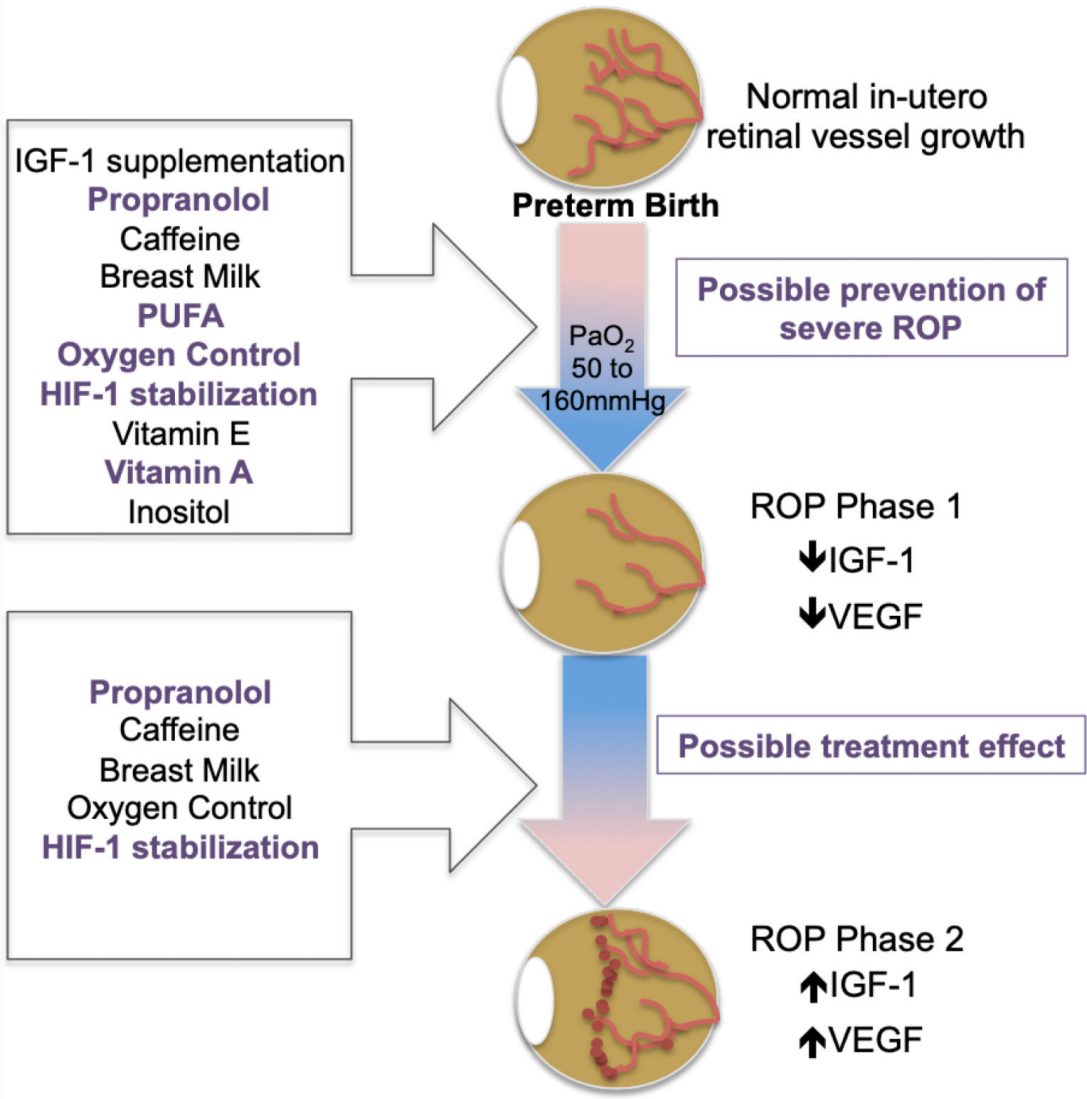
Preventive strategies target post-natal molecular pathogenesis

As depicted in Figure 2, current ROP prevention strategies, based on known post-natal ROP patho-mechanisms, have failed to demonstrate singular efficacy for disease prevention. In some cases treatments have resulted in deleterious effects. Therefore, additional approaches are necessary to more effectively achieve early intervention that enables us to alter the natural course of disease and promote normal retinal vascularization in the ex-utero environment. While, as some authors have postulated, this may be achieved through combined effects from current strategies that show incomplete benefit, it may also be true that understanding events “up-stream” of post-natal mechanisms may best inform novel prevention strategies.

#### A role for maternal and placental contributions in ROP disease

ROP is typified by a multifactorial etiology with most of our current investigation directed at genetic and environmental risk. Certainly, numerous candidate genes have been identified, including brain derived neurotropic factor, and key factors involved in the canonical Wnt signaling pathway, such as frizzled class receptor-4 and low density lipoprotein receptor-related protein 5. However, none of the allelic variants identified thus far sufficiently impact ROP pathogenesis to inform novel screening, prevention or treatment paradigms as reviewed recently by Swan et al., [150]. The contributions of maternal and placental pathobiology to preterm infant ROP development are under-studied by comparison and thus, poorly understood. Despite, the strong (0.73) heritability factor of ROP predicted from twin studies [10, 105, 151, 152], preliminary work suggests that maternal and placental physiology can influence preterm infant ROP risk in meaningful ways. The placenta is the primary maternal/fetal interface and functions to exchange nutrients and oxygen between the mother and infant. The full complement of injury following deprivation of placental support in preterm birth is not clear, as placental function evolves during fetal maturation. While it is accepted that abrupt loss of placental support is deleterious to global infant development in the immediate postnatal period [153] as exemplified in the Barker hypothesis, long-term health outcomes are also affected by maternal and placental pathobiology including cardiovascular, diabetic and neurologic disease [154–156]. Therefore, an understanding of the maternal and placental fetal support factors lost at the time of preterm birth may provide insight into novel early disease mechanisms, better enabling preterm infant disease prevention.

There is precedent for the influence of placental pathobiology on post-natal preterm infant diseases including bronchopulmonary dysplasia, necrotizing enterocolitis, neurocognitive and neurovascular development [157–163]. Furthermore, preterm infant inflammatory and neurotrophin protein levels at birth, thought to be mediated in-utero by the placenta, are associated with brain volumes and cognition [164]. Preliminary work in ROP similarly has shown that elevated levels of inflammatory proteins in preterm infant systemic circulation at birth is associated with increased ROP risk, whereas a decreased risk of ROP was associated with higher levels of angiogenic and neurotrophic factors [165]. Moreover, a study of CpG methylation profiles in DNA from the placentas of mothers with Extremely Low Gestational Age Newborns (ELGAN) study [166] showed that CpG methylation variability corresponded to infant systemic protein level changes associated with ROP risk [167]. Finally, placental function has been shown to inform fundamental ROP pathological mechanisms, including systemic preterm infant IGF-1 expression [168]. Thus, there is precedent and preliminary evidence for the potential role of maternal and placental pathobiology informing ROP pathogenesis.

#### A novel model of natural ROP protection

While simply understanding the influence of maternal/placental physiology and pathobiology on preterm infants may provide a valuable framework for understanding ROP risk and pathological mechanisms, doing so within a natural model of placentally-mediated ROP protection may add further insight into potential therapeutic approaches. This idea is supported by evidence showing that placental pathologies associated with preterm birth, particularly those with intra-uterine infection/inflammation such as chronic or acute chorioamnionitis are generally strongly associated with increased ROP risk [105, 169–171]. However emerging evidence suggests that maternal preeclampsia may represent an exception with respect to ROP risk and severity, and may even represent a natural model of ROP protection, with potential to provide novel understanding of early protective ROP mechanisms. There is considerable support for this relationship in the epidemiologic literature, including recent systematic reviews and meta-analyses [18, 172–179]. Several studies dispute this protective relationship [180–182], or find no association [183], however, these studies are largely limited by small sample size and lack of appropriate controls (GA, BW and presence of preterm labor) and most have suggested larger analyses are needed. The largest studies, up to 25K in one study [172], reproducibly demonstrate ROP protection in preeclampsia. Further, we know that ethnicity, NICU care (i.e. oxygen targets), and infant GA at birth influence the measured affect. This is highlighted in a recent publication by Shulman et al. [176], who found that preterm infants with GA and BW risk for ROP (< 32 weeks and 1,500 g) demonstrated ROP protection with preeclampsia; however, infants with no GA or BW risk for ROP, GA at birth ~38 weeks, had an increased co-occurrence of ROP and preeclampsia. Thus, it is important to consider the population demographics when analyzing the relationship between preeclampsia and ROP. These data are further supported by epidemiologic data in other forms of placental insufficiency demonstrating ROP protection [184]. Shulman et al. [176], further suggest that prematurity, while necessary for ROP risk, may confound the measured association between maternal preeclampsia and ROP. Certainly the etiologic basis for preterm delivery is varied in most populations, particularly the relatively smaller population delivering in the ROP risk window prior to 32 weeks gestation. It is possible that the mechanisms informing spontaneous preterm delivery as opposed to induced preterm delivery (as is often the case in maternal preeclampsia) predispose to greater risk of ROP rather than preeclampsia predisposing to lesser ROP risk. This question remains to be answered in humans, although we are beginning to address it with studies controlling for the presence of labor, which demonstrate no ROP effect from preterm labor as opposed to induced or spontaneous (i.e. cervical inconvenience, etc.) preterm delivery [10, 17]. This is supported by rodent studies from Becker et al. [185], in which premature birth was uncoupled from uteroplacental insufficiency (UPI, a major component of preeclampsia) using a rat uterine artery ligation model. They found that in the setting of term gestation, uteroplacental insufficiency resulted in a significant decrease in avascular area and a trend towards reduced proliferative vascularization in the OIR model of ROP, supporting a relationship between pr ROP from non-preeclamptic spontaneous preterm births-it can still be argued that mechanisms underlying reduced likelihood of ROP development in the setting of preeclampsia will inform approaches toward normalizing retinal vascular development and ROP prevention. Taken together, understanding mechanisms of ROP protection in preeclampsia is a novel strategy to identify earlier ROP risk, informed by maternal and placental pathobiology, which may allow for more efficacious in-utero or postnatal prevention.

There are varied hypotheses regarding mechanisms of ROP protection in the setting of preeclampsia. Preeclampsia is a complex maternal-fetal-placental pathology typified by placental insufficiency of unclear molecular basis; however, a recent systems biology analysis of preeclampsia confirmed prior work and suggested a more comprehensive maternal etiology for preeclampsia [186]. Therefore, mechanisms contributing to preeclampsia, and thereby ROP protection in this setting, are likely multifactorial. Multiple lines of evidence in the literature suggest that placental pathobiology is an underlying factor in our current paradigm of ROP pathogenesis. For example, Becker et al. [185], suggest that increased systemic angiogenic factors are the etiologic basis for ROP protection in their OIR rat model. Although this has been incompletely assessed in humans, preeclampsia in humans involves dysregulation of angiogeneic factors, particularly in the maternal circulation [187–189]. Work from other groups has linked placental function to fatty acid deficit and ROP risk. Macronutrient transport protein expression has been associated with cellular-level placental histologic changes in preeclampsia; specifically, increased fatty acid placental transfer and maternal systemic circulation have been suggested to mediate disease in early onset preeclampsia [190]. As discussed previously, ROP development has been associated with decreased circulating fatty acids longitudinally from birth [191]. Combined, these studies suggest that an integrated systems approach, combining maternal and placental pathobiology relative to preterm infant ROP can elucidate underlying mechanisms of protection that can be therapeutically replicated post-natally or even in-utero to prevent ROP.

While placental evaluation is possible in human disease and demonstrates the greatest translational value, manipulation of risk variables and local tissue (retinal) biochemical and genomic analysis is prohibitive. Preeclampsia/placental insufficiency can be modeled in rodents and ROP outcomes subsequently assessed using the OIR model of ROP. The “gold standard” approach for placental insufficiency is uterine artery ligation, which directly modulates blood flow to the placenta, thereby achieving insufficiency. While, as noted previously, this model of placental insufficiency does demonstrate decreased severity of pre-retinal neovascularization in the OIR model, there are limitations to this approach including the extreme degree and abrupt onset of hemodynamic insufficiency, which likely does not fully recapitulate preeclamptic pathophysiology now believed to be a systemic condition [186]. Alternative models, including chronic systemic delivery of a thromboxane A2 analog which is commonly overproduced in preeclampsia, represents a more systemic model of preeclamptic pathophysiology and thus, may better inform relative to the human condition [192–194].

## Conclusions

**Figure 3.**
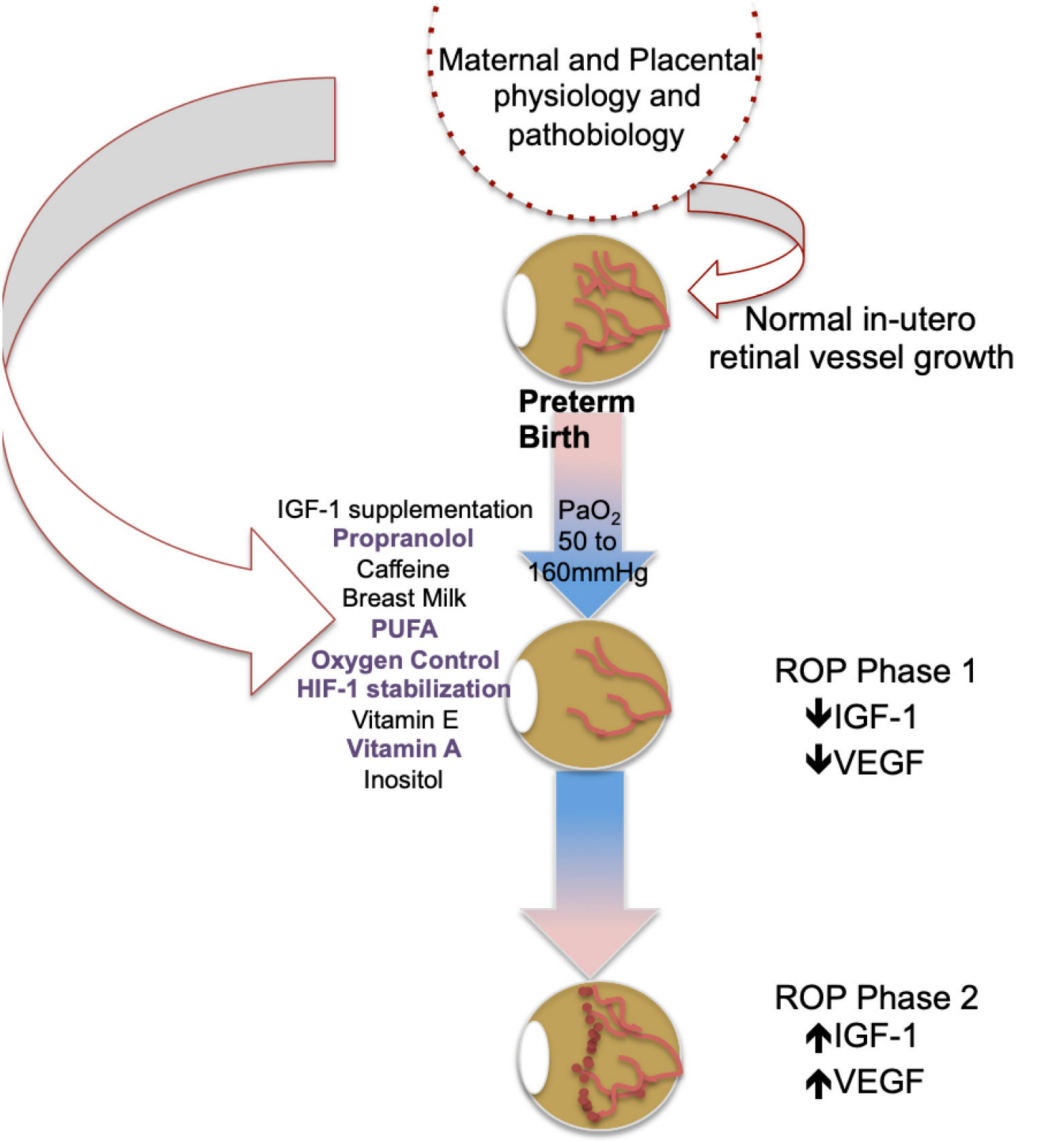
Maternal and placental-informed ROP preventive

ROP has significant clinical impact on the life-long vision of preterm infants, even in the setting of mild disease. Our treatments are only indicated in the most severe presentations and are associated with significant visual comorbidity and possible deleterious systemic effects. We currently lack the ability to meaningfully prevent ROP disease and facilitate normal retinal vascularization. While numerous strategies have been tested, to date none have demonstrated sufficient efficacy for complete ROP prevention. Emerging evidence suggests an important role for maternal and placental physiology and pathobiology in preterm infant post-natal and lifelong disease burden, including ROP risk and mechanisms. As depicted in Figure 3, preliminary work suggests a role for maternal and placental contributions to physiologic in-utero mechanisms of normal retinal development as well as known mechanisms informing the current paradigm of ROP pathogenesis. The contribution of placental function to subsequent infant ROP disease is further highlighted in preeclamptic placental insufficiency, which paradoxically demonstrates ROP protection. These contributions are “upstream” of our current understanding and thus may allow for earlier disease prediction and intervention, prior to substantial vessel loss or neovascular retinal disease. We hypothesize that greater understanding of maternal and placental contributions in both the physiologic and pathologic conditions will allow us to advance our understanding of early ROP risk and patho-mechanisms and design novel strategies for not only ROP prevention but also facilitation of normal retinal vascularization for all preterm infants.

## Abbreviations

BM: breast milk
BW: birth weight
DHA: docosahexaenoic acid
EPA: eicosapentaenoic acid
GA: gestational age
HIF-1: hypoxia inducible factor-1
IGF-1: insulin growth factor-1
NSAID: non-steroidal anti-inflammatory drug
OIR: oxygen induced retinopathy
PUFAs: polyunsaturated fatty acids
rhIGF-1: recombinant human IGF-1
rhIGFBP-3: recombinant human IGF binding protein-3
ROP: retinopathy of prematurity
VEGF: vascular endothelial growth factor
